# The Role of Subjective and Objective Social Status in the Generation of Envy

**DOI:** 10.3389/fpsyg.2020.513495

**Published:** 2020-12-15

**Authors:** Henrietta Bolló, Dzsenifer Roxána Háger, Manuel Galvan, Gábor Orosz

**Affiliations:** ^1^Doctoral School of Psychology, Eötvös Loránd University, Budapest, Hungary; ^2^Institute of Psychology, ELTE Eötvös Loránd University, Budapest, Hungary; ^3^Institute of Cognitive Neuroscience and Psychology, Research Centre for Natural Sciences, Hungarian Academy of Sciences, Budapest, Hungary; ^4^Faculty of Humanities and Social Sciences, Institute of Psychology, Pázmány Péter Catholic University, Budapest, Hungary; ^5^Department of Psychology, University of North Carolina at Chapel Hill, Chapel Hill, NC, United States; ^6^Univ. Artois, Univ. Lille, Univ. Littoral Côte d’Opale, ULR 7369 - URePSSS - Unité de Recherche Pluridisciplinaire Sport Santé Société, Sherpas, France

**Keywords:** malicious envy, benign envy, subjective social status, objective social status, deservingness

## Abstract

Envy is a negative emotion experienced in response to another person’s higher status. However, little is known about the composition of its most important element: status. The present research investigates the two main forms of social status (objective and subjective) in the generation of envy. In Study 1, participants recounted real-life situations when they felt envious; in Study 2 we examined whether the effect was the same in a controlled situation. We consistently found that those who were the most respected in the eyes of others were envied more than the richest ones. Furthermore, perceived deservingness of the superior other’s success differentiated between benign and malicious envy. Although previous studies focused on material comparisons when investigating envy, our results indicate that envy is rather a subjective social status related emotion. Not material, but social advantage of the superior other causes the most painful envy and future studies should put more emphasis on this type of social comparison in envy research.

## Introduction

Envy is a status-related painful emotion (Steckler and Tracy, 2014), however, little is known about how envy is related to the two main forms of social status by considering its subjective and objective aspects. Previous studies on envy focused more on the objective aspects, like income inequalities (Fiske, 2011), and different purchases (Lin et al., 2018), but less research focused on the subjective side of social status.

Both objective and subjective social status (SSS) are relevant to many psychological constructs, such as mental health (Franzini and Fernandez-Esquer, 2006), negative affectivity, pessimism, stress, control over life, active and passive coping (Adler et al., 2000), well-being (Howell and Howell, 2008), depressive symptoms (Hoebel et al., 2017), and the probability for experiencing shame (Lundberg et al., 2009), and pride (Bolló et al., 2018).

Individuals with high SSS receive respect and admiration from others and exert significant influence in their social groups (e.g., family, workplace, and friends, etc.). In contrast, individuals with low SSS receive no respect or admiration, and have limited influence in their important social groups (Shaked et al., 2016). SSS is therefore inherently based on social consensus (Anderson et al., 2015). In contrast to SSS, objective social status (OSS) refers to tangible resources, material possessions, and educational background. OSS is usually measured by income, financial wealth, education, type of home, household goods, and type of car (Adler and Stewart, 2007).

There is not always a clear link between SSS and OSS (Centers, 1949). Individuals with low OSS do not necessarily think of themselves as inferior because they may have a high SSS in their social groups. These people experience respect and admiration despite their low OSS. At the same time, those with the highest salaries or newest cars (high OSS) may feel unappreciated and disrespected if they do not exert influence in important social groups (low SSS). In summary, OSS refers to material goods, while SSS refers to social factors.

According to the *affect as information hypothesis* (Schwarz and Clore, 2003), emotions function to inform and navigate individuals in a social hierarchy. Humans evolved to navigate within complex social structures, and as a result, they must be capable of quickly responding to different social cues about their status relative to others (Sedikides and Skowronski, 1997; Robins et al., 1999). Emotions underlying these social dynamics serve to increase the stability of social hierarchies and avoid costly disputes (Steckler and Tracy, 2014).

Envy is a negative emotion experienced in response to another’s higher status. According to the social-functional approach to envy, the goal of envy is to lessen the social status gap between the self and a superior other (Van de Ven et al., 2009; Lange and Crusius, 2015a,b). There are two qualitatively distinct forms of envy, which motivate different behaviors. Malicious envy drives people to lower the status of a superior other, while benign envy motivates individuals to increase their own status, often by increasing personal effort (Salovey and Rodin, 1984; Lange and Crusius, 2015a). Malicious envy is associated with hostility, destructive social consequences, and resentful thoughts, whereas benign envy entails more positive thoughts toward the envied person (Van de Ven et al., 2009).

An important difference between benign and malicious envy is the perceived deservingness of the superior other’s success (Van de Ven et al., 2009, 2012; Crusius et al., 2017). It is important to mention that deservingness is different from entitlement. In a workplace situation, a colleague may be entitled to a promotion based on the number of years he/she has worked for the company but may not deserve it (Van de Ven et al., 2012). Deservingness refers to earned outcomes while entitlement refers to lawful outcomes (Feather, 2003).

As deservingness is one of the main topics in envy research, it is important to define what variables determine people’s judgements on deservingness. One of the key variables that influences whether or not an individual is seen to deserve the consequences of his/her behavior is the attribution of personal responsibility (Hareli and Weiner, 2002). According to the theory of causal attributions, an outcome is seen as deserved if the individual is responsible for it and undeserved if the outcome is unintended due to uncontrollable causes with either external or internal causes (Feather, 1992). For example, a student who gets a good grade on an exam because he/she picked up a lucky topic is seen as less responsible and the success is more likely to be seen as undeserved, contrasted to a student who studied for months.

However, Feather (1992) emphasized that, besides responsibility, two other variables have to be taken into account to determine deservingness: values and justice. The value analysis implies that there is a certain class of situations where the outcome that follows controllable and intentional behavior can be undeserved. In more detail, an outcome is perceived to be undeserved if a negatively valued behavior (for example dishonest practices) is followed by a positive outcome (such as good grades) or vice versa, a positive behavior (for example hard work) is followed by a negative outcome (such as failing an exam). These are unbalanced structures according to Heider’s (1958) theory. In contrast, balanced structures are more likely to be perceived as deserved (Feather, 1992).

Focusing on the dynamics between deservingness and envy, when individuals perceive the superior other’s success as undeserved, the subjective feeling of injustice can result in hostile tendencies which promote malicious envy (Smith, 1991). Earlier research on envy suggested that envy originally involves the sense that the envied person’s advantage is undeserved (Smith et al., 1994). However, early studies conceptualized only malicious envy as “envy proper,” because they differentiated types of envy based on the presence or absence of hostile behavioral tendencies (Smith and Kim, 2007); recent theories differentiate types of envy based on the motivational consequences (Lange and Crusius, 2015a).

Although there is a clear link between undeservingness and the hostile component of envy, being envious about someone else’s deserved advantage can help to explain why envy motivates both a desire to hurt the superior other and to do better (Schaubroeck and Lam, 2004; Cohen-Charash, 2009). If the superior other’s success is considered as deserved, people tend to think that hard work pays off, and they will have the motivation to emulate the superior other. This will then promote benign envy (Van de Ven et al., 2012; Lange and Crusius, 2015a).

Regarding the link between the intensity of envy and deservingness there are two main theories. On the one hand, the undeserved advantage of the superior other can lessen the intensity of (undifferentiated) envy due to the fact that it is easier to accept that the other is just better (Ben-Ze’ev, 1990). On the other hand, Miceli and Castelfranchi (2007) suggest the opposite direction, that deserved advantage of the other person causes more intense envy, because deserved advantage makes individuals’ demerits appear more salient and distressing, and therefore threatening for self-esteem. In their empirical study, Van de Ven et al. (2012) found no evidence for such a link between deservingness and the intensity of envy but rather the differentiating effect of deservingness on the types of envy.

Recently, scholars have started to examine the underlying mechanisms of what triggers envy, but the results are still contradictory. A study by Lin et al. (2018) found that posting experiential purchases (e.g., traveling) on social network sites triggered more envy than posting material purchases (e.g., new car). On the other hand, some scholars suggest that as material purchases are easier to compare, they are more likely to generate social comparisons and therefore elicit envy (Smith and Kim, 2007; Carter and Gilovich, 2010).

Thus, as there are some contradictions regarding what triggers envy, we propose to take a step back and investigate the composition of the status difference between the self and the superior other. Both material and experiential purchases are indicators of another person’s OSS, as they both depend on money, therefore previous studies on envy neglected the other main form of status, namely SSS. In the present research we investigated the differentiated role of OSS and SSS in the generation of envy. We hypothesized that SSS is a more relevant construct, as factors related to our identity may cause the most painful frustration, which is the most fundamental element of envy (Salovey and Rodin, 1984; DeSteno and Salovey, 1996). In previous research on the role of SSS and OSS in the generation of pride, Bolló et al. (2018) found that SSS played a more prominent role and individuals overestimated the relevance of material possessions in hypothetical situations. As pride is in a social-functional relation with envy (Lange and Crusius, 2015b), it is reasonable to suppose a similar pattern of SSS in the generation of envy. Furthermore, contrary to previous studies, we included the existing knowledge about the role of deservingness as it may have a modulating effect between status and type of envy (Van de Ven et al., 2012; Crusius and Lange, 2017). We also differentiated between benign and malicious envy.

## Study 1

In Study 1 we investigated the effect of social status on envy by asking participants to recall real-life situations. We also tested the role of deservingness, as this is the primary appraisal dimension that differentiates between benign and malicious envy (Van de Ven et al., 2012; Crusius and Lange, 2014). In light of previous research (Bolló et al., 2018; Lin et al., 2018), we predicted that benign envy would be higher when SSS was deserved than when it was undeserved, and than when OSS was deserved or undeserved. Moreover, we predicted that malicious envy would be higher when SSS was undeserved than when it was deserved, and than when OSS was deserved or undeserved. In other words, deservingness should have a reverse effect on benign and malicious envy for SSS but there should be no such difference for OSS.

### Methods

#### Participants

A total of 399 Hungarian participants were recruited from topic-irrelevant social media groups with more than 10,000 members. Of these, 345 were female and all were aged between 18 and 65 (*M*_age_ = 32.41 years, *SD*_age_ = 11.69 years). As far as their level of education was concerned, 305 of them had a university degree (76.4%), 88 (22.1%) had finished high school, and six (1.5%) had finished elementary school. A total of 143 (35.8%) lived in Budapest, 193 (48.4%) lived in towns, and 63 (15.8%) lived in small towns or villages.

#### Materials and Procedure

Participants first gave their informed written consent in accordance with the Declaration of Helsinki by ticking a box before taking part in the online study. Participants were taken straight to the end of the survey if they did not give this consent. Participants were then randomly assigned to one of two conditions, OSS or SSS.

In the OSS condition participants were asked to think of a friend, colleague, or acquaintance who has better material circumstances than they do (e.g., has more money, has more financial security, has a nicer home, or has a better car). Participants were asked to answer the following questions in writing: “How long have you known each other?,” “How did you meet?,” “What is your relationship with this person like?,” and “Name something this person has which you want more of.”

In the SSS condition, participants were asked to think of a friend, colleague, or acquaintance who they deem to have more respect, admiration, and influence in the eyes of others. They were then asked to write responses to the same questions as those given to the OSS group.

The participants were then asked to complete the BeMaS Scale (Lange and Crusius, 2015a), which assesses levels of benign and malicious envy. Although the BeMaS is designed to measure dispositional envy, in this study it was adapted to measure envy of a particular person. The scale consists of ten items, a benign subscale of five items (e.g., “If I notice that this other person is better than me, I try to improve.”; α = 0.766), and a malicious subscale of five items (e.g., “I want this other person to lose his/her advantage”; α = 0.861). Participants were asked to describe their envious feelings toward this previously identified superior other on a scale from 1 (does not apply at all) to 6 (applies very much). Afterward, participants were asked to indicate whether they perceived the identified superior other’s advantage as deserved or undeserved. The final questions elicited demographic information. All materials are available here: https://osf.io/7u3y4/.

#### Data Analysis

The effects of status and deservingness (as a quasi-experimental variable) on envy were analyzed using the Generalized Linear Mixed-effect Model (GLMM, IBM SPSS 22). In the model the fixed effects included status (OSS vs. SSS), deservingness (undeserved vs. deserved), and type of envy (benign vs. malicious), and each participant’s ID was included as a random factor. All possible two-way and three-way interactions of the fixed factors were tested. Statistical tests were two-tailed and the α value was set at 0.05. Sequential Sidak correction was applied in all *post hoc* pairwise comparisons. All statistics were performed using IBM SPSS Statistics 21.

### Results

**FIGURE 1:**
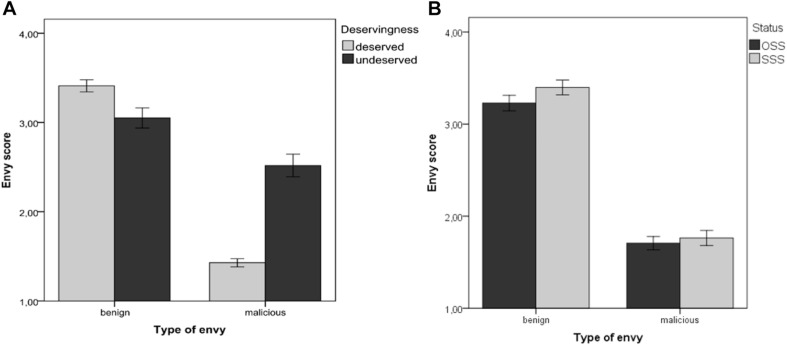
**(A)** The interaction between deservingness and type of envy in Study 1 and **(B)** Difference of envy types between OSS and SSS conditions. Error bars represent one standard error of the mean.

There was no significant difference between the two conditions (OSS and SSS) regarding gender, age, place of residence, and educational level (all *p*-values > 0.05). The GLMM analysis showed that status had a significant main effect on envy, *F*(1, 790) = 4.51, *p* = 0.03, $\eta_{\text{p}}^{2}$ = 0.01, and indicating higher envy ratings for SSS (M_SSS_ = 2.58, SE_SSS_ = 0.07) than OSS (M_OSS_ = 2.46, SE_OSS_ = 0.07). Furthermore, there was significant interaction (Figure 1) between deservingness and type of envy: *F*(1,790) = 85.422, *p* < 0.001, and $\eta_{\text{p}}^{2}$ = 0.0.10. Pairwise comparison revealed that benign envy was more likely if the superior other was perceived to have a deserved advantage (M_deserved_ = 3.41, SE_deserved_ = 0.06) than if it was deemed to be undeserved (M_undeserved_ = 3.06, SE_undeserved_ = 0.10), *t*(790) = 2.930, *p* = 0.003, 95% CI (0.116; 0.586), and malicious envy was more likely if the superior other was perceived to have an undeserved advantage (M_undeserved_ = 2.56, SE_undeserved_ = 0.10) than if it was deemed to be deserved (M_deserved_ = 1.43, SE_deserved_ = 0.06), *t*(790) = 9.43, *p* < 0.001, 95% CI (−1.37; −0.89). All statistics for the fixed effects and their interactions can be found in Supplementary Table 1.

### Discussion

Study 1 demonstrated that envy is more intense when the superior other is better off socially than when he/she has more material possessions. One potential explanation may be that comparisons in relation to SSS have a higher degree of self-relevance to individuals than material ones (Lin and Utz, 2015; Lin et al., 2018). In other words, individuals become more envious when they feel that they have less respect and influence among certain people than a superior other does. Individuals are less envious of a superior other’s money and possessions, and previous research has demonstrated that individuals overestimate the importance of these possessions (Bolló et al., 2018; Lin et al., 2018).

Furthermore, our results provide further empirical evidence for the link between deservingness and the type of envy that is generated (Van de Ven et al., 2012; Crusius et al., 2017). If the advantage of the imagined superior other was considered deserved, it was more likely to elicit benign envy. If the envier is outperformed by someone who is in fact better off, he/she will become frustrated and will increase efforts to be similar (Salovey and Rodin, 1984; Lange and Crusius, 2015a). However, if the envier is outperformed by someone perceived as undeservedly better off, he/she will become frustrated, but the subjective feeling of injustice will promote hostile tendencies (Smith, 1991).

In summary, Study 1 provides evidence to support the hypothesis that SSS plays a more prominent role in the generation of envy. However, in Study 1 the comparative reference point was chosen by the participants, therefore the individual differences of the social distance with the superior could distort the results. Furthermore, participants needed to rely on personal memories that can differ in reliability. There could also be a discrepancy between real-time and retrospective evaluations of unpleasant memories (Redelmeier and Kahneman, 1996), which could be caused by the limitations of human memory capacity and other social-cognitive abilities, like imagining a concrete picture of the acquaintance. Recalling such complex memories like in Study 1 can be overwhelming for participants and lead to biased results. Therefore, in Study 2 we decided to give a standard reference point in order to investigate the role of status in the generation of envy to investigate the role of status in a controlled vignette situation by systematically manipulating SSS and OSS, where participants did not need to recall personal memories.

## Study 2

In Study 2, we systematically manipulated social status and perceived deservingness in a hypothetical situation against a standard reference point. We predicted that within SSS, deservingness should have opposite effects on benign and malicious envy. On the other hand, in contrast to Study 1, we expected that the effect of deservingness on OSS would be similar, as individuals tend to overestimate OSS in hypothetical situations (Bolló et al., 2018; Lin et al., 2018).

### Methods

#### Participants

A total of 389 Hungarian participants were recruited from topic-irrelevant social media groups with more than 10,000 members. Of these, 332 were female and all were aged between 18 and 64 (*M*_age_ = 31.74 years, *SD*_age_ = 11.77 years). As far as their level of education was concerned, 296 (76.1%), of them had a university degree, 85 (21.9%) had finished high school, and eight (2.1%) had finished elementary school. A total of 132 (33.9%) lived in Budapest, 193 (49.6%) lived in towns, and 64 (16.5%) lived in small towns or villages.

#### Materials and Procedure

Participants gave their informed written consent in accordance with the Declaration of Helsinki by ticking a box before participating in the online study. Participants were taken straight to the end of the survey if they did not give this consent.

A 2 × 2 vignette study was carried out to investigate the effects of social status (OSS or SSS) and perceived deservingness (deserved or undeserved) on benign and malicious envy.

Following procedures similar to those in Study 1, participants were asked to imagine that they had been working for a multinational company and that “Gabi” (which is a gender neutral name in Hungarian) was one of their colleagues. Participants were asked to imagine a scenario in which their OSS and SSS were average. Gabi was superior either in terms of OSS or SSS and the status was either deserved or undeserved. OSS was characterized by financial situation, education, phone, type of home, and clothes. SSS was characterized by the level of respect, admiration, and influence among colleagues. An example of higher deserved OSS was a better financial situation because of hard work. An example of higher undeserved OSS was a better financial situation because Gabi had “cozied up” to the boss. An example of higher deserved SSS was Gabi commanding more respect, admiration, and influence among other colleagues because Gabi is dependable. Higher undeserved SSS was characterized by more respect, admiration, and influence among others because Gabi had “cozied up” to everyone.

Participants were then asked to complete the BeMaS Scale (Lange and Crusius, 2015a), which measures benign and malicious envy. Participants were asked to describe how they would feel about “Gabi” using the same procedure as in Study 1 (e.g., for benign envy: “I would strive to reach Gabi’s superior achievements,” α = 0.774. For malicious envy: “Seeing Gabi’s achievements would make me resent him/her,” α = 0.825). Finally, questions were asked in relation to participants’ gender, age, education, and place of residence.

In Study 2 we did not ask for a manipulation check for the following reasons based on Hauser et al. (2018). First, questions on deservingness after reading the quite short vignettes could have an effect on the participants’ thinking by reflecting information about the researcher’s hypothesis and whether that variable was supposed to affect the answers to the following questions. Second, answering questions where respondents can express their dislike can lessen the intensity of their emotions by the time they get to the main dependent emotion variable. Asking respondents to describe their feelings right after the manipulation can help them to connect their feelings more strongly to the eliciting event. On the other hand, drawing respondents’ attention to undeservingness and crystalizing the unfairness of the situation can intensify negative feelings. All materials are available here: https://osf.io/7u3y4/.

### Results

There was no significant difference between the four groups regarding gender, age, place of residence, and educational level (all *p*-values > 0.05).

**FIGURE 2:**
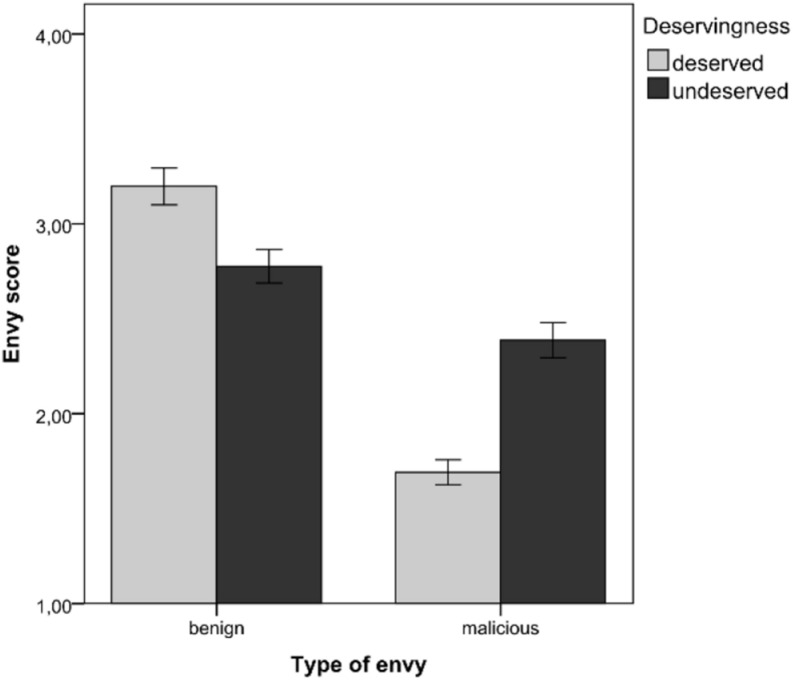
The interaction between deservingness and type of envy in Study 2. Error bars represent one standard error of the mean.

The GLMM analysis showed that status had a significant main effect on envy, *F*(1, 770) = 5.63, *p* = 0.018, $\eta_{\text{p}}^{2}$ = 0.01, and which indicates higher levels of envy in relation to SSS (M_SSS_ = 2.63, SE_SSS_ = 0.07) than to OSS (M_OSS_ = 2.40, SE_OSS_ = 0.06). Furthermore, there was significant interaction (Figure 2) between deservingness and the type of envy, *F*(1, 770) = 59.56, *p* < 0.001, and $\eta_{\text{p}}^{2}$ = 0.07. Pairwise comparison revealed that benign envy was more likely if the superior other was deemed to have a deserved advantage (M_deserved_ = 3.20, SE_deserved_ = 0.09) than if the advantage was perceived to be undeserved (M_undeserved_ = 2.78, SE_undeserved_ = 0.09), *t*(770) = 3.407, *p* = 0.001, and 95% CI (0.177; 0.658). Malicious envy was more likely if the superior other was deemed to have an undeserved advantage (M_undeserved_ = 2.39, SE_undeserved_ = 0.09) than if the advantage was perceived to be deserved (M_deserved_ = 1.69, SE_deserved_ = 0.09) *t*(770) = 5.665, *p* < 0.001, and 95% CI (0.454; 0.935). All statistics for the fixed effects and their interactions can be found in Supplementary Table 2.

### Discussion

Study 2 gave further empirical evidence for our hypothesis regarding the prominent role of SSS in the generation of envy. Accordingly, if someone else is more respected and better off in a social sense it generates more painful envy. Although, Study 2 was contradictory to previous findings (Bolló et al., 2018; Lin et al., 2018), suggesting that people are prone to overestimate OSS in hypothetical vignette situations. In the present vignette study, individuals did not confer more importance to OSS. However, there are some differences from previous studies. In the study by Lin et al. (2018), the superior others were the respondents. Furthermore, in the study of Bolló et al. (2018) it was also the respondents’ own OSS and SSS that were compared.

Furthermore, as in Study 1 and previous research (Van de Ven et al., 2012; Lange and Crusius, 2015a), Study 2 provided further empirical evidence for the differentiating role of deservingness. Our results confirmed that perceptions of deservingness are linked to benign envy (Salovey and Rodin, 1984; Lange and Crusius, 2015a) while perceptions of undeservingness are linked to malicious envy (Smith, 1991).

## General Discussion

According to the social-functional approach to envy, the goal of envy is to lessen the social status gap between the self and a superior other (Van de Ven et al., 2009; Lange and Crusius, 2015a,b). Previous research on envy was more focused on material inequalities (Carter and Gilovich, 2010; Fiske, 2011; Lin and Utz, 2015; Lin et al., 2018); the present research aimed to investigate the subjective facet of social status as well, taking into account the role of deservingness. Our findings suggest that SSS intensifies feelings of envy more than OSS and that deservingness helps differentiate between benign and malicious envy. A potential explanation for the prominent role of SSS in envy is that social factors are more related to our identity and cause more frustration, which can result in envy (Salovey and Rodin, 1984; DeSteno and Salovey, 1996; Lin et al., 2018). It has to be noted that previous studies (Salovey and Rodin, 1984; DeSteno and Salovey, 1996) confirmed this relationship in the case of jealousy, although recent research gave empirical evidence for the role of self-relevance in the case of envy as well (Lin et al., 2018). Although envy and jealousy have some overlap regarding hostility, lowered self-esteem, and sadness, they are two distinct emotions (Parrott and Smith, 1993). Envy is more focused on inferiority and therefore can be characterized by self-diminishment and resentment, while jealousy is more focused on the threat of loss of another’s fidelity and can be characterized by anxiety, distrust, and anger (Parrott and Smith, 1993).

There are two contradicting theories regarding the role of material things in envy. Some scholars suggest that, as material possessions are easily comparable, individuals compare themselves more frequently in this domain, and that consequently envy is experienced more in relation to material possessions (Carter and Gilovich, 2010). In contrast, others suggest that envy is most intense when social comparison is important for a person’s identity (Salovey and Rodin, 1984; Bolló et al., 2018).

Furthermore, although previous studies indicated that individuals tend to exaggerate the importance of OSS in hypothetical situations (Bolló et al., 2018), this study did not confirm this finding. In Study 2 respondents were asked to evaluate their feelings in a hypothetical situation, but SSS still played a more prominent role. However, in Study 2 respondents were asked to imagine that they were in the role of the envier, while in previous studies they were either the envied one (Lin et al., 2018) or the comparison affected their own status (Bolló et al., 2018). The findings of this study therefore suggest that there is a discrepancy between what individuals believe others are envious of and what they themselves are envious of, which can be a direction for future research.

Furthermore, the present research study replicated previous findings about the role of deservingness in envy (Parrott and Smith, 1993; Lange and Crusius, 2015b; Crusius and Lange, 2017; Crusius et al., 2017). Benign envy was more likely to be expressed when the superior other’s outcome was deserved and malicious envy was more likely when it was seen to be (Study 1) or characterized as (Study 2) undeserved. In the present research we applied the value theory of deservingness by Feather (1992). By definition, deservingness refers to whether the outcome is contingent with the situation: if there is a fit between the situation and the outcome it is deserved, otherwise it is undeserved (Feather, 1999). Based on Feather (1992), in Study 2 we characterized deserved advantage by positively valued behaviors (hard work in the case of OSS and being dependable in the case of SSS) and undeserved advantage by a negatively valued behavior (“cozying up” to others) and both were followed by the same positive outcome. In the present study, attributing the superior other’s success as undeserved (negatively valued behavior followed by a positive outcome) promoted malicious envy, while attributing the other’s advantage as deserved (positively valued behavior followed by a positive outcome) promoted benign envy. According to attribution theory (Hareli and Weiner, 2002), balanced structures (Heider, 1958) carry the possibility of controllability (namely that hard work pays off) and individuals will have the motivation to work hard and become as successful as the superior other.

In summary, the findings indicate that SSS and OSS play different roles in the generation of envy. SSS is more relevant in upward social comparisons leading to benign and malicious envy, and material possessions do not motivate people to move up the social hierarchy to the same extent.

## Limitations and Future Studies

Although this study has important implications in relation to envy, there are a number of limitations which should be taken into account. Firstly, females were over-represented in the sample, which may lead to biased results. Previous studies suggest that women are more likely to avoid socially comparative situations that men (Niederle and Vesterlund, 2007; Rand, 2017), which can have an effect on envy.

Secondly, both studies were cross-sectional and no behavioral measures were used. Future studies should apply longitudinal or experimental design with behavioral measures.

Thirdly, Study 2 was a situation evaluation task with an imaginary scenario, so participants’ reactions in this imaginary situation may differ from their reactions in a real-life scenario. Although applying a vignette method in Study 2 can lead to interesting and informative contributions, there are limitations, especially for examining potentially less desirable emotions like envy (Van Dijk et al., 2006). For example, in the undeserved conditions of Study 2 participants might draw negative evaluations of not only the outcome but also the person themselves and it might also affect their answer about envy, added to “deservedness,” as Crusius and Lange (2014) pointed out previously that malicious envy biases attention toward the envied person rather than the advantage of this person. In future studies, this bias can be treated with manipulating not only the deservingness of the outcome but also the characteristics of the person, and then investigating their interactions.

However, vignette studies have long been used in experimental emotion research, offering the possibility to systematically control for other factors by providing identical information to respondents, thereby increasing their internal validity (Powell et al., 2008). In addition, there is empirical evidence that vignette studies can be highly generalizable to real life behavior, while overcoming the ethical, practical, and scientific limitations associated with alternative methods (Evans et al., 2015). Furthermore, respondents were assured about their anonymity and were encouraged to answer honestly. In sum, despite the limitations of online hypothetical methods, they are widely used in envy research (for example, Parrott and Smith, 1993; Lange and Crusius, 2015b; Poelker et al., 2019) and there is also empirical evidence that people do not seem more reluctant to report envy than other negative social emotions (Hareli and Weiner, 2002).

There are several potential directions for future studies, but the most important is that more emphasis should be placed on social factors instead of material inequalities in envy research. SSS is a broad conception but investigating its elements, such as respect or influence, could be a fruitful area. Furthermore, future studies should investigate possible mediating variables between envy and social status. Some possible mediators may be status maintenance strategies, prestige, and dominance. Previous research indicates that prestige is related to SSS (Bolló et al., 2018) and benign envy (Crusius and Lange, 2017).

## Conclusion

The social function of envy is to lessen the status gap between the self and a superior other, but little is known about the nature of this status. This study aimed to investigate the role of OSS and SSS in the generation of envy. We found that SSS intensifies envy more than OSS and that deservingness plays a differentiating role.

## Data Availability Statement

All data and materials are available here: https://osf.io/7u3y4/.

## Ethics Statement

The studies involving human participants were reviewed and approved by Research Ethics Committee of Eötvös Loránd University’s Faculty of Education and Psychology. The patients/participants provided their written informed consent to participate in this study.

## Author Contributions

HB made substantial contributions to conception and design. HB and DH were involved in the acquisition of data and contributed to analysis and interpretation of data. HB drafted the article. All authors revised the article critically for important intellectual content and final approval of the version to be published, agreed to be accountable for all aspects of the work in ensuring that questions related to the accuracy or integrity of any part of the work are appropriately investigated and resolved by all authors.

## Conflict of Interest

The authors declare that the research was conducted in the absence of any commercial or financial relationships that could be construed as a potential conflict of interest.
