# Supplementary material for: The Role of Subjective and Objective Social Status in the Generation of Envy
Source: Front Psychol. 2020 Dec 15;11:513495. doi: 10.3389/fpsyg.2020.513495 (PMC7770237; doi:10.3389/fpsyg.2020.513495)
Supplement: Supplementary file 1 [file Table_1.docx]

**Supplemental Information**

The role of subjective and objective social status in the generation of envy

Henrietta Bolló^1,2,3^, Dzsenifer Roxána Háger^4^, Manuel Galvan^5^, Gábor Orosz^6^

^1^ Doctoral School of Psychology, Eötvös Loránd University, Budapest, Hungary

^2^ Institute of Psychology, ELTE Eötvös Loránd University, Budapest, Hungary

^3^ Institute of Cognitive Neuroscience and Psychology, Research Centre for Natural Sciences, Hungarian Academy of Sciences, Budapest, Hungary

^4^ Faculty of Humanities and Social Sciences, Institute of Psychology, Pázmány Péter Catholic University, Budapest, Hungary

^5^ Department of Psychology, University of North Carolina at Chapel Hill, Chapel Hill, NC, United States

^6^ Univ. Artois, Univ. Lille, Univ. Littoral Côte d’Opale, ULR 7369 - URePSSS - Unité de Recherche Pluridisciplinaire Sport Santé Société, Sherpas, France

Table 1S. GLMM results of Study1

| *Fixed factors* | F (df1, df2) | *Sig.* | ƞ_p_^2^ |
| --- | --- | --- | --- |
| Deservingness | 19.07 (1, 790) | < .001 | 0.03 |
| Status | 4.51 (1, 790) | .034 | 0.01 |
| Type of envy | 240.32 (1, 790) | < .001 | 0.23 |
| Deservingness × Status | 1.69 (1, 790) | .204 | 0.00 |
| Deservingness × Type of envy | 85.42 (1, 790) | < .001 | 0.10 |
| Status × Type of envy | 0.44 (1, 790) | .507 | 0.00 |
| Deservingness × Status × Type of envy | 2.95 (1, 790) | .086 | 0.00 |

Table 2S. GLMM results of Study2

| *Fixed factors* | F (df1, df2) | *Sig.* | ƞ_p_^2^ |
| --- | --- | --- | --- |
| Deservingness | 1.95 (1, 770) | .16 | 0.00 |
| Status | 5.63 (1, 770) | .02 | 0.01 |
| Type of envy | 173.68 (1, 770) | < .001 | 0.18 |
| Deservingness × Status | 2.72 (1, 770) | .10 | 0.00 |
| Deservingness × Type of envy | 59.57 (1, 770) | < .001 | 0.07 |
| Status × Type of envy | 2.32 (1, 770) | .13 | 0.00 |
| Deservingness × Status × Type of envy | 0.79 (1, 770) | .37 | 0.00 |
